# Occupancy-Based Energy Consumption Estimation Improvement through Deep Learning

**DOI:** 10.3390/s23042127

**Published:** 2023-02-14

**Authors:** Mi-Lim Kim, Keon-Jun Park, Sung-Yong Son

**Affiliations:** 1 Department of Next Generation Smart Energy System Convergence, Gachon University, Seongnam-si 13120, Republic of Korea; 2 Smart Energy System Convergence Research Institute, Gachon University, Seongnam-si 13120, Republic of Korea

**Keywords:** occupancy, deep learning, energy consumption, building energy, estimation improvement

## Abstract

The energy consumed in buildings constitutes more than half of the total electricity consumption and is highly correlated with the number of occupants; therefore, it is necessary to use occupancy information in energy consumption analysis. However, the number of occupants may not be accurate owing to measurement errors caused by various factors, such as the locations of sensors or cameras and the communication environment. In this study, occupancy was measured using an object recognition camera, the number of people was additionally collected by manual aggregation, measurement error in occupancy count was analyzed, and the true count was estimated using a deep learning model. The energy consumption based on occupancy was predicted using the measured and estimated values. To this end, deep learning was used to predict energy consumption after analyzing the correlation between occupancy and energy consumption. Results showed that the performance of occupancy estimation was 1.9 based on RMSE, which is a 71.1% improvement compared to the original occupancy sensing. The RMSE of predicted energy consumption based on the estimated occupancy was 56.0, which is a 5.2% improvement compared to the original energy estimation.

## 1. Introduction

### 1.1. Background of Research

Buildings account for more than 55% of global electricity consumption, and this figure is increasing on average by 1.1% annually owing to improved living standards, unusual climates, and increased indoor dwelling time [1]. Building energy consumption is mainly influenced by climate, building cover, building services and systems, operation and maintenance, occupants’ activities and behavior, and indoor environmental quality [2]; in addition, there exists research on the method of estimating occupancy (detection and estimation of occupancy) and how user characteristics and activities relate to the energy consumption of buildings [3]. However, occupancy measurement errors are caused by external factors, sensors, camera installation locations, and communication environments. This actual information error will affect the actual energy consumption prediction performance. Therefore, it is necessary to improve the prediction of energy consumption by improving the accuracy of occupancy estimation.

### 1.2. Related Work

In this regard, a study on the methods for estimating occupancy information (occupancy estimation and detection) and analysis of energy consumption based on occupancy information is in progress. To estimate occupancy information, occupancy data must be measured and collected using passive infrared (PIR) sensors, carbon dioxide (CO_2_) sensors, wireless fidelity (WiFi), and camera images. A PIR sensor can detect occupants by detecting changes in the infrared radiation of an object. It has the advantages of low cost, low energy consumption, high reliability, and environmental adaptability; however, it only provides binary information, and the accuracy varies depending on the distance between the sensor and the occupant [4,5]. A CO_2_ sensor can detect changes in indoor CO_2_ concentration and collect occupancy information. Although the cost is low, the accuracy of the sensor depends on the opening of windows and doors as well as the installation location [5]. A WiFi device can estimate occupancy by measuring the MAC address and RSSI of smart mobile devices (MDs) using the existing WiFi infrastructure. However, using this information to estimate occupancy is affected by the WiFi communication environment—whether the MDs are connected to the WiFi, whether the occupants have MDs, and how many MDs the occupants have [5]. A camera using a vision sensor similar to those of the human eye can detect occupancy changes with high accuracy but is affected by lighting conditions, lower camera quality, and privacy issues [6].

Each of these sensors has advantages and disadvantages, and the disadvantages are being researched to overcome them [5,6]. Raykov et al., implemented an occupancy estimation system using an infinite hidden Markov model (iHMM) based on a PIR sensor [7]. Lee et al., estimated occupancy using camera images to overcome the problem of a PIR sensor that cannot detect stationary objects. A convolutional neural network (CNN), which is an artificial intelligence (AI) technology, was used for this with a recognition rate of 85% [8]. Zuraimi et al., estimated occupancy using both a machine learning approach (artificial neural networks (ANNs), prediction error minimization, and support vector machines (SVM)) and dynamic mass balance based on a CO_2_ sensor. As a result of the experiment, the average root mean square error (RMSE) was 12.5 [9]. Ouf et al., estimated occupancy using multiple regression analysis after collecting CO_2_ sensor and WiFi connection data and analyzed the correlation between occupancy data recorded directly on-site. The coefficient of determination (R2) was 0.530 in the case of CO_2_-based occupancy estimation and 0.703 for WiFi-based occupancy estimation, and when CO_2_ and WiFi were used together, R2 was 0.792 [10]. Alishahi et al., collected occupancy data using a WiFi network management platform and camera, analyzed the correlation, and predicted day-ahead occupancy using k-means and multiple regression analysis (MLR) [11]. Wang et al., estimated occupancy using a large-scale conservation equation based on a CO_2_ sensor used at dawn or when illumination was less than a certain amount, and they estimated the camera-based occupancy during the day with an accuracy of 91% [12]. Ravi et al., proposed an algorithm for estimating the occupancy by matching occupancy counts measured using a camera and WiFi connection counts by area. In the case of WiFi-alone estimation, there was an average error of 63.4%, and in the case of camera-based estimation, there was an average error of 25.3%; however, the error was reduced to an average of 16.2% by applying the estimation algorithm [13]. Wang et al., collected environmental sensor data and WiFi connection data and then confirmed the correlation and error between the data collected using the adaptive snare model and actual occupancy data collected by the camera. Subsequently, the occupancy was estimated with all possible combinations using an ANN. As the number of sensor types increase, the estimation error can increase [14].

In previous studies, occupancy estimation was performed using single or multiple sensors. In particular, a camera was used to collect sensing data to estimate occupancy or to collect the actual measurement data to analyze occupancy. Camera-based occupancy measurement utilizes AI technology to recognize people; therefore, measurement errors can occur when it is used for sensing data. Therefore, this study proposes a method for estimating occupancy using deep learning by analyzing the measurement error of a camera equipped with object recognition technology.

Occupancy information has been applied in various studies related to energy saving and optimal operations for efficient energy utilization in buildings. Simma et al., analyzed the energy-saving effects of HVAC systems through simulations using occupancy information [15]. Salimi et al., developed an optimization model that could operate an HVAC system optimally based on occupancy information [16]. Shi et al., developed an MPC algorithm that could control HVAC systems based on occupancy information [17]. Tekler et al., analyzed the energy-saving effect by remotely controlling the plug load based on occupancy information [18].

In addition, studies related to occupancy information have been conducted using various approaches to estimate and predict the energy consumption of buildings. Ding et al., estimated the occupancy based on a PIR sensor and predicted the energy consumption (lights, plug, and HVAC) using a probability function and Markov model. The prediction results of the occupancy-based model did not exceed 5% of the actual energy consumption [19]. Wei et al., proposed a model for estimating occupancy based on a CO2 sensor and predicted energy consumption (HVAC) using neural network models (feed-forward neural network (FFNN), elaboration likelihood model (ELM), and ensemble). The energy prediction performance was 0.92, on average, when occupancy was not considered and 0.93 when occupancy was considered [20]. Markovic et al., proposed an approach to predict the day-ahead energy consumption (plug) using long short-term memory (LSTM). The proposed model was applied to buildings other than the target building without correction; the mean relative error (MRE) was reduced by approximately 5% when considering occupancy data based on motion sensors [21]. Anand et al., predicted energy consumption using a nonlinear energy prediction model (support vector regression (SVR), random forest (RF), gradient boosting (GB), artificial neural network feed-forward (ANN-FF), deep network-based artificial neural network (ANN-DN), and conventional multiple regression (MR)) using occupancy data based on the number of WiFi connections. As for the energy consumption prediction performance, the average mean absolute error (MAE) was 29.79 based on the validation dataset, and the ANN-DN model’s performance was 23.6, which was superior to other models [22]. Wang et al., proposed a model to predict energy consumption (plug) by considering occupancy. The LSTM network was used to predict energy consumption, and 95% of the predicted results were within ±1 kW of the actual energy consumption. The energy prediction error was reduced by 5–6% by considering the occupancy data collected by the camera-based sensor installed at the entrance [23]. Anand et al., estimated occupancy based on a camera and proposed a model for predicting energy consumption based on occupancy using multiple nonlinear regression (MNLR) and deep neural network (DNN)-based algorithms. Using the proposed model, the energy-saving potential was an average of 4.43% for plug load and 49.03% for lighting load [24]. Table 1 shows the prediction model and error results from previous studies. The results are summarized based on RMSE, which is commonly used in these studies. However, the performance values in the table do not imply the relative excellence of the prediction models because the load types, sizes, and sensors of each study are different.

As previous studies have found, energy consumption prediction performance is improved when occupancy is considered. However, occupancy information is different from actual occupancy owing to measurement errors caused by external factors, the installation location of sensors or cameras, and the communication environment. Therefore, the prediction of energy consumption must be improved by estimating the occupancy as described above.

### 1.3. Proposed Method

Therefore, this study estimates occupancy using a deep learning model and proposes a method to improve the energy consumption prediction performance based on deep learning using the estimated occupancy. First, occupancy data were collected using a camera equipped with AI object detection technology and energy consumption data were collected using a smart meter. Then, measurement errors from the camera were analyzed using the collected occupancy data, and occupancy was estimated using deep learning. Finally, after an analysis of the correlation between occupancy and energy consumption, the energy consumption was predicted by deep learning based on the measured and estimated occupancy, and the proposed method was compared and evaluated.

The remainder of this paper is organized as follows: Section 2 presents the methodology and includes methods for collecting occupancy and energy consumption data, estimating occupancy, and predicting energy consumption. Section 3 analyzes the experimental results according to the experimental environment. Finally, Section 4 provides the conclusion and discusses future work.

## 2. Methodology

**Figure 1:**
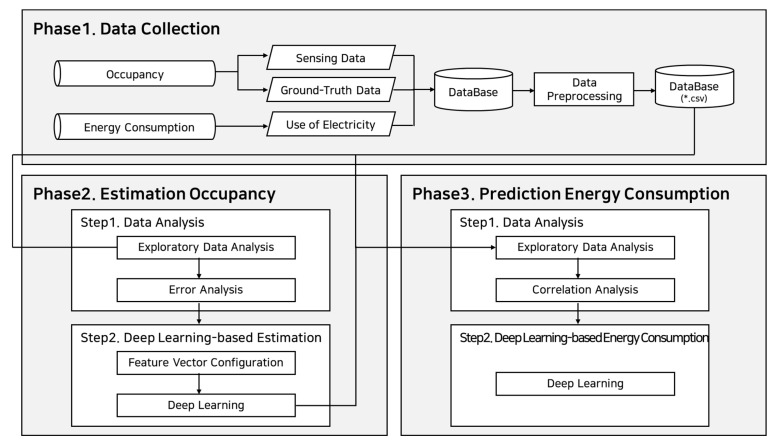
Methodology for estimating occupancy and predicting energy consumption using deep learning.

As shown in Figure 1, this study proposes a methodology consisting of three steps for occupancy estimation and energy consumption prediction. The first phase (Phase 1) was the collection and preprocessing of the occupancy and energy consumption data. The second phase (Phase 2) was the analysis of the measured and estimated occupancy, and the final phase (Phase 3) was the analysis of energy consumption data and the prediction of energy consumption based on the occupancy.

### 2.1. Data Collection

#### 2.1.1. Data Collection for Occupancy

As mentioned in a previous study, with improved camera recognition, cameras have been used recently to collect occupancy data [7]. To measure occupancy, a camera equipped with object recognition technology was used. However, accurate occupancy measurement based on a camera with object reorganization was still difficult because it was accompanied by measurement errors owing to the performance of the object recognition technology installed in the camera. Therefore, in this study, basic occupancy was measured with a camera (type-1 camera) used for object recognition, and for reliable measurement, occupancy was measured using an Internet protocol camera (type-2 camera). The type-1 camera recognized objects in real time, stored the occupancy counts in its own database, and automatically or manually transmitted them to the integrated database. The occupancy data collected by the type-1 camera were used as the sensing data. The type-2 camera recorded images in real time and stored them in its database. To measure occupancy from the stored camera image, a person manually checked the camera image and counted the number of occupants. The occupancy counts were recorded only for events with occupancy changes and were then saved as a csv file in the integrated base. Occupancy data collected using a type-2 camera were used as ground-truth data.

#### 2.1.2. Data Collection for Energy Consumption

To predict energy consumption based on occupancy, data on the consumption of electricity, which is the main energy source of a building, were collected. Electricity consumption data are composed of HVAC, light, and plug loads, and only the plug loads most closely related to occupancy are collected using a smart meter [11,12]. Collected data were stored in an integrated database.

#### 2.1.3. Data Preprocessing

Because the data were collected non-uniformly owing to different measurement times and communication environment problems, they were stored after preprocessing in a new database. Although the sensing data from the type-1 camera were collected in real time, the measured time was different for each installed device, and hence these data were preprocessed through interpolation of the previous value at a time interval of a second. Because the ground-truth data from the type-2 camera were recorded only when a change in occupancy occurred, preprocessing was performed with data at a time interval of a second through interpolation of subsequent values.

Moreover, the energy consumption data were collected as accumulated electricity consumption data at a time interval of a second; therefore, the accumulated data were converted into energy consumption data at a time interval of a minute, and preprocessing was performed through linear interpolation for outliers and missing values.

### 2.2. Methodology for Estimating Occupancy

#### 2.2.1. Data Analysis for Estimating Occupancy

Sensing data collected using a type-1 camera contain sensing errors, and therefore data analysis is required through comparison with ground-truth data collected using a type-2 camera. First, an exploratory analysis was performed through visualization of the time-series data of the collected sensing data and ground-truth data to analyze the characteristics of the collected occupancy data and observe the error characteristics based on time. Subsequently, the error characteristics of the sensing data were analyzed through the relationship between the sensing data and the ground-truth data to analyze the error distribution for the occupancy count. In addition, camera-based sensing data have measurement errors caused by the surrounding environment (light, occlusion, etc.), and a representative situation related thereto was analyzed. Then, because it is difficult to analyze the occupancy count error at a time interval of a second for all general situations, the distribution characteristics were analyzed using a histogram by resampling the data at a time interval of a minute. The analyzed distribution characteristics were used to estimate occupancy based on deep learning.

#### 2.2.2. Estimating the Occupancy Based on Deep Learning

This section presents the development of a deep learning-based occupancy estimation model by composing histogram information into feature vectors to minimize the error in occupancy counts using sensing data. To learn the deep learning model, the occupancy count of sensing and ground-truth data were used as input data; in addition, time information was used in consideration of the sensor being affected by the collection environment and living patterns of occupants when sensing data. As described in the previous section, sensing data collected in real time are converted into the frequency of occupancy count in minutes using a histogram to construct a feature vector, and the feature vector including date, hour, and minute date time information is constructed. The feature vector is expressed by Equation (1) as follows:

(1) $$X=\left(d,h,m,h_{0}\ldots h_{m}\right),$$

where X, d, h*,* and *m* denote the feature vector, date, hour, and minute, respectively. $h_{0}$ represents the frequency of occupancy count when the occupancy count is zero, and $h_{m}$ represents the frequency of occupancy count when the maximum occupancy count is m. The output vector was composed of ground-truth data at a time interval of a minute through the mode value of the corresponding time. The feature vector is normalized using a standard normal distribution (Gaussian distribution) and expressed in Equation (2) as follows:

(2) $$Z=\frac{X-\mu }{\sigma }.$$

**Figure 2:**
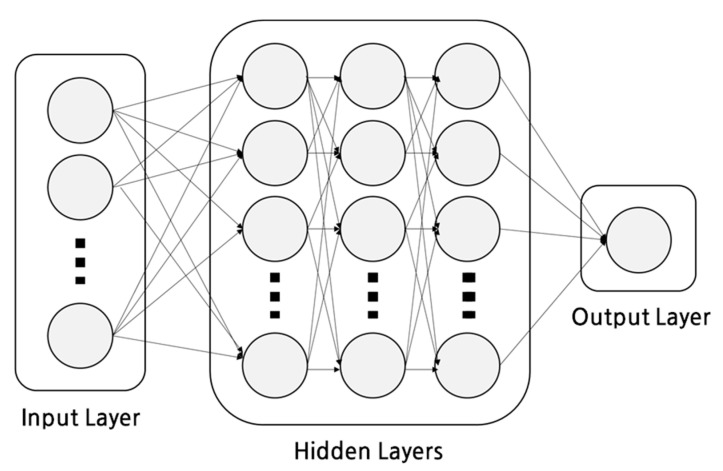
DNN structure.

An ANN is a learning algorithm that mimics the human brain’s neural network and comprises an input layer, hidden layers, and an output layer. As the number of hidden layers increases, the ANN has a problem of poor learning and overfitting owing to the gradient vanishing problem. DNN emerged as the advanced ANN owing to the introduction of various techniques, such as various activation functions, parameter initialization, and regulation methods, and the improvement of hardware performance [25]. The model used in this study is a DNN with a five-layer structure consisting of an input layer, four hidden layers, and one output layer, as shown in Figure 2.

During DNN operation, the input signal of the input layer is transmitted to the hidden layer, and the received input signal is calculated as the sum of the connection weights in the hidden layer and the output through the activation function. The signal output from the hidden layer enters the input of the next hidden layer and is calculated and output in the same manner. The last layer, the output layer, receives the output of the last hidden layer as the input and is calculated as the sum of the weights. The final output of the DNN is expressed by Equation (3), as follows:

(3) $$y=\tau (w^{T}\cdots \tau \left(w^{T}\tau \left(w^{T}x\right)\right)$$

where x represents the input, $w^{T}$ represents the weight, and τ represents the activation function. The activation function uses the exponential linear unit (eLu) model, which is the same as Equation (4) [26].

(4) $$\begin{array}{c} \tau \left(x\right)=x,\;\;if\;x>0\\ \;\;\tau \left(x\right)=\sigma \left(e^{x}-1\right),\;\;if\;x\leq 0 \end{array}$$

The error in the value predicted by the model was quantified by the loss function, and the weight of each node was updated so that the error was minimized during training. The loss function is based on MSE. Because the result estimated by the DNN is for occupancy, it was postprocessed as a positive integer. To evaluate the performance of the DNN model, the error between the measured and estimated occupancies was compared and evaluated. For this purpose, MAE, MSE, and RMSE were used as performance indices, as shown in Equations (5)–(7).

(5) $$MAE=\frac{1}{n}\overset{n}{\underset{i=1}{\sum }}\left|y_{i}-{\hat{y}}_{i}\right|$$

(6) $$MSE=\frac{1}{n}\overset{n}{\underset{i=1}{\sum }}{\left(y_{i}-{\hat{y}}_{i}\right)}^{2}$$

(7) $$RMSE=\sqrt{\frac{1}{n}\overset{n}{\underset{i=1}{\sum }}{\left(y_{i}-{\hat{y}}_{i}\right)}^{2}}$$

### 2.3. Methodology for Estimating Energy Consumption

#### 2.3.1. Data Analysis for Estimating Energy Consumption

Energy consumption is greatly influenced by occupancy, as mentioned in previous studies. Therefore, energy consumption must be analyzed together with occupancy. First, energy consumption data were visualized for time-series characteristics together with ground-truth data on occupancy to perform an exploratory analysis. Thus, the characteristics of the collected energy consumption were analyzed. Subsequently, the relationship between energy consumption and occupancy was analyzed. The occupancy data consist of sensing data, ground-truth data, and data estimated using deep learning performed as described in the previous section. The relationship between occupancy and energy consumption was analyzed using the Pearson correlation coefficient (PCC). Based on the analyzed correlation results, the energy consumption based on the occupancy was predicted and comparatively analyzed.

#### 2.3.2. Estimating Energy Consumption Based on Deep Learning

This section describes the development of a deep learning-based energy consumption prediction model by configuring the occupancy count as a feature vector to predict energy consumption [27]. In deep learning model learning, time information was also used, and the energy consumption was affected by the occupants’ living patterns. A feature vector was constructed, including occupancy count and time information about the date, hour, and minute, and the feature vector is shown in Equation (8).

(8) Z=(d,h,m,o),

where Z, *d*, *h*, *m*, and *o* denote a feature vector for energy consumption prediction, the date, the hour, the minute, and the occupancy count, respectively. The output vector comprises the energy consumption. The feature vector for energy consumption prediction was normalized using the normal distribution in Equation (2). The DNN model with the same structure introduced in the previous section was used for energy consumption prediction; however, a rectified linear unit (ReLu) was used for the activation function. The activation function ReLu is expressed in Equation (9) as follows [28]:

(9) τ(x)=max(0,x)

In addition, the performance of the developed energy consumption prediction model was evaluated based on the MAE, MSE, and RMSE, given in Equations (5)–(7), respectively, as well as the CV (RMSE) (Equation (10)) and MAPE (Equation (11)).

(10) $$CV\left(RMSE\right)=\frac{n}{\sum_{i=1}^{n}y_{i}}\sqrt{\frac{\sum_{i=1}^{n}{\left(y_{i}-{\hat{y}}_{i}\right)}^{2}}{n}}$$

(11) $$MAPE=\frac{1}{n}\overset{n}{\underset{i=1}{\sum }}\left|\frac{y_{i}-{\hat{y}}_{i}}{y_{i}}\right|$$

The developed energy consumption prediction model can predict and analyze three types of occupancy.

## 3. Experiments and Results

### 3.1. Experimental Environment

**Figure 3:**
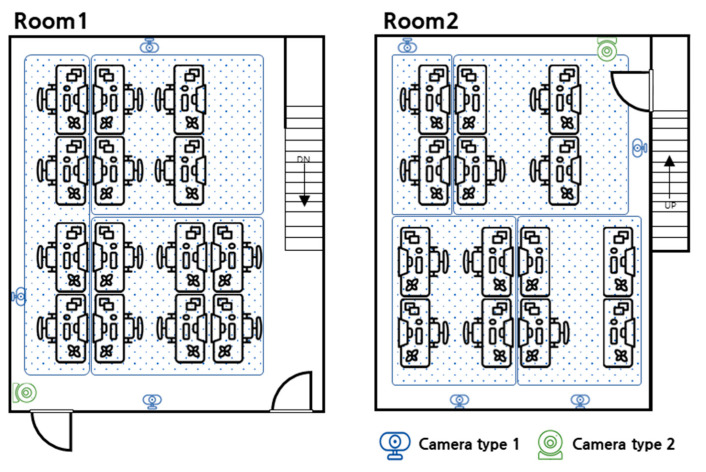
Room structure and camera installation location and area of case study.

For the estimation of occupancy and the prediction of energy consumption, data from the measured occupancy and electricity consumption were collected. Two rooms of the Smart Energy System Convergence Research Institute located at Gachon University in Seongnam, Gyeonggi-do, Republic of Korea, were selected. The two rooms were composed of two floors, as shown in Figure 3. The first and second floors had 14 and 10 seats, respectively, and each room was used by doctoral, master’s, and undergraduate students. Data were collected during working hours (9:00 to 18:00) over an eight-day period. Because the collection was during a vacation period, graduate students stayed on the premises, except for meetings and other external schedules, and undergraduate students were often absent because there were no classes.

**Figure 4:**
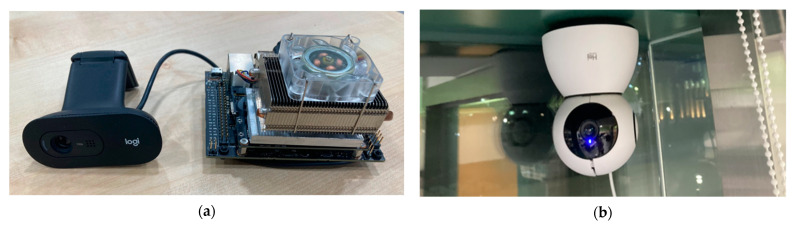
Camera for collecting occupancy data: (**a**) camera for sensing data collection (type-1); (**b**) camera for ground-truth data collection (type-2).

Two types of cameras were used, as shown in Figure 4. A type-1 camera was used to collect the sensing data using an NVIDIA Jetson Nano equipped with SSD Inception v2, a Linux-based developer board, which is a single-step detection method for recognizing objects [29]. The GPU of the board was a 128-core NVIDIA Maxwell, the CPU was a Quad-core ARM A57 @ 1.43 GHz, and the memory was 2GB 64-bit. Object recognition was performed every 5 s, and the numbers of recognized people were collected as sensing data using a WiFi network. In the case of type-2 camera, images were recorded in real time with an IP camera, and to accurately measure the occupancy, the occupancy was manually counted from the recorded images and used as the ground-truth data.

Because a type-1 camera measures the occupancy count through person recognition, various situations that interfere with person recognition may occur, such as the situation when a person is far away or obscured. Therefore, it is difficult to measure occupancy using a single camera. As shown in Figure 3, three cameras were installed on the first floor and four cameras were installed on the second floor. A type-2 camera was installed with one camera at the entrance of the first and second floors and was used to accurately measure occupancy by checking the number of people entering.

**Figure 5:**
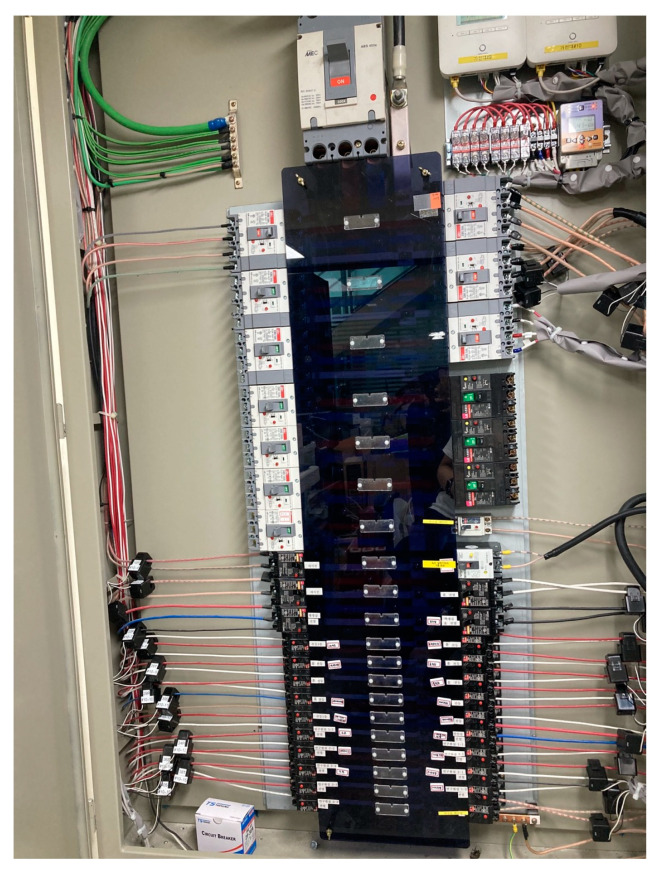
Smart meter installed for energy consumption data collection.

For energy data collection, a current transformer (CT) and potential transformer (PT) were installed in the distribution box, as shown in Figure 5. The collected data were transmitted to a data concentration unit (DCU) for convenience of data processing, and serial and Ethernet communication was used. Energy data were measured five times per second on average and collected as the accumulated electricity consumption. Electricity consumption was measured because the plugs in rooms 1 and 2 and the air conditioner of the server room were connected to each other.

Sensing data collected through the type-1 camera were preprocessed at a time interval of a second through interpolation of previous values. Ground-truth data collected by the type-2 camera were preprocessed at a time interval of a second through the interpolation of subsequent values.

Energy data were not collected for an average of 2 min per day owing to a problem with the inspection of the device, and this part was preprocessed through linear interpolation. Additionally, the accumulated energy consumption during the collection period was converted into energy consumption at a time interval of a minute.

To estimate occupancy, a five-layer DNN consisting of four hidden layers with 512, 256, 64, and 32 neurons, respectively, and one output layer with one neuron was used. The batch size was 32, and an eLu activation function, batch normalization, and weight initialization (He initialization) were used for all layers. Adam optimization was used for parameter optimization, and the learning rate was set to 0.02. For energy prediction, a four-layer DNN consisting of three hidden layers with 128, 64, and 32 neurons, respectively, and one output layer with one neuron was used. Hyperparameter settings were similar to those in the actual residence estimation model; the activation function was ReLU, and the optimized learning rate was set to 0.03. The epochs of the actual room occupancy estimation and energy consumption prediction were 200, but the patience was set to 10 to prevent overfitting so that it could terminate early. The experiment was repeated 20 times to ensure data reliability.

### 3.2. Estimation of Occupancy

#### 3.2.1. Data Analysis

**Figure 6:**
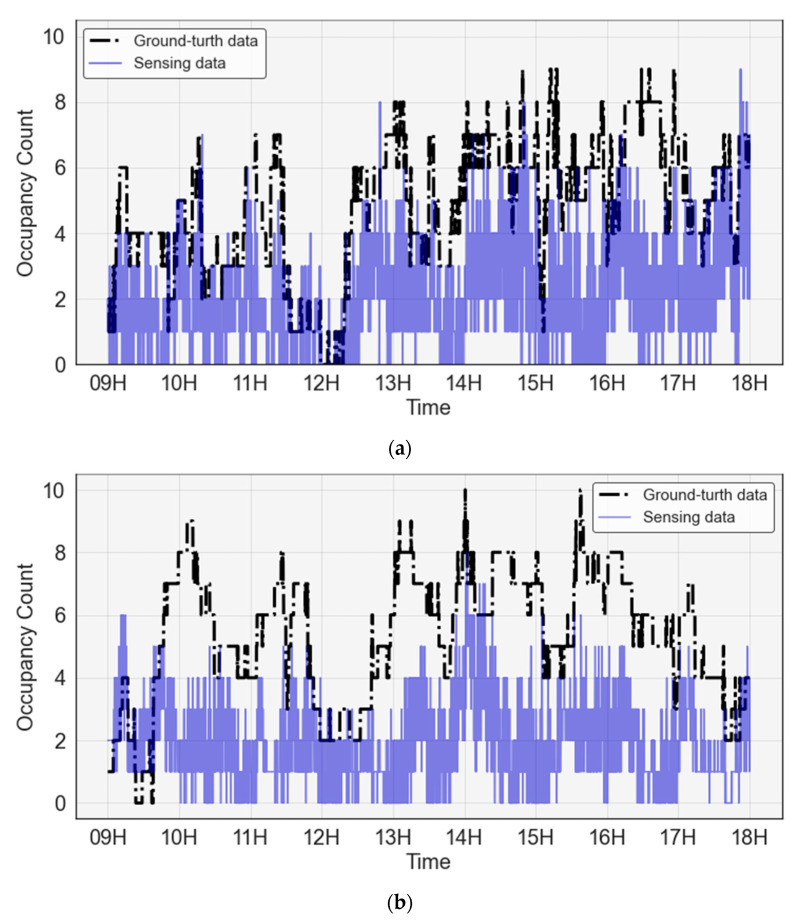
Sensing and ground-truth occupancy count data: (**a**) room 1; (**b**) room 2.

In this section, data analysis performed on the sensing data and ground-truth data on the number of occupants collected in Phase 1 is explained. Figure 6 shows the occupancy data collected at 1 s time intervals for one day during the entire collection period for rooms 1 and 2. The blue solid line represents the sensing data and the black dotted line represents the ground-truth data. The occupancy pattern based on the time of rooms 1 and 2 showed that occupancy increased at approximately 9:00, the commute time for both rooms 1 and 2, and decreased during lunchtime. Subsequently, the occupancy increases again from around 13:00 and decreases again after 17:00, the evening and time of leaving. In addition, room 1 showed more people and frequent movements than room 2. In particular, because of the nature of the room structure, people in room 2, located on the second floor, tended to go outside after talking with the people in room 1 on the first floor, and this was observed at around 17:00.

**Figure 7:**
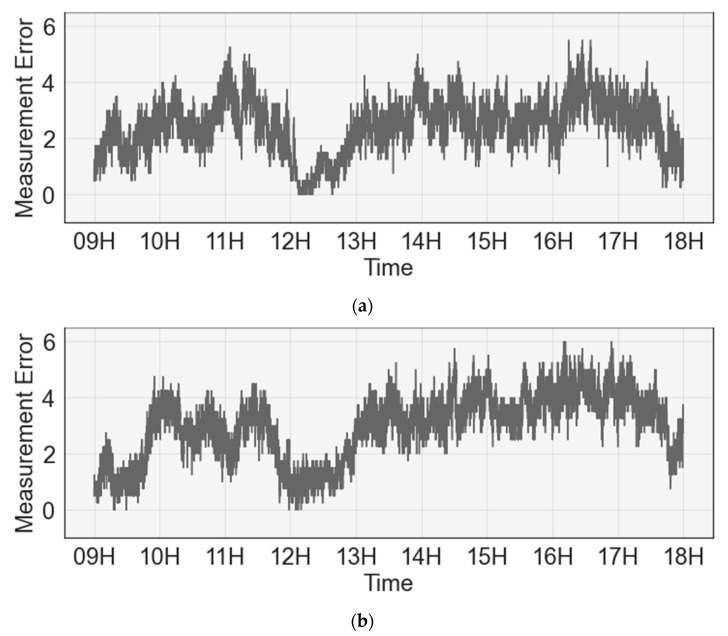
Analysis of time-zone error between sensing and ground-truth data: (**a**) average measurement error in room 1; (**b**) average measurement error in room 2.

Figure 7 shows the average error of the occupancy count of the sensing data during the collection period of rooms 1 and 2 through a time-series plot. The *X*-axis represents time, and the *Y*-axis represents the average error between the sensing and ground-truth data. Measurement error occurs in approximately three to four people in the intervals 9:00–10:00 and 12:00–13:00 and in approximately one person in the intervals 10:00–12:00 and 13:00–17:00.

**Figure 8:**
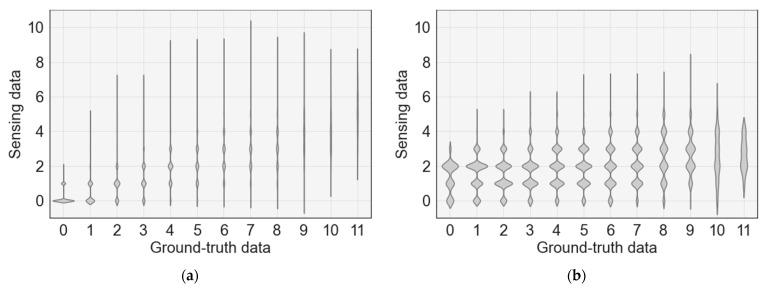
Analysis of occupancy count error distribution between sensing and ground-truth data: (**a**) occupancy distribution in room 1; (**b**) occupancy distribution in room 2.

Figure 8 shows the error distribution of the occupancy count between the ground-truth and sensing data of rooms 1 and 2. The *X*-axis represents the ground-truth data and the *Y*-axis represents the sensing data. In both rooms 1 and 2, as the ground-truth data value increased, the sensing data value also tended to increase; however, the occupancy count tended to be underestimated overall. In particular, when the occupancy in both rooms 1 and 2 is more than 10, it can be observed that the occupancy error is slightly underestimated.

**Figure 9:**
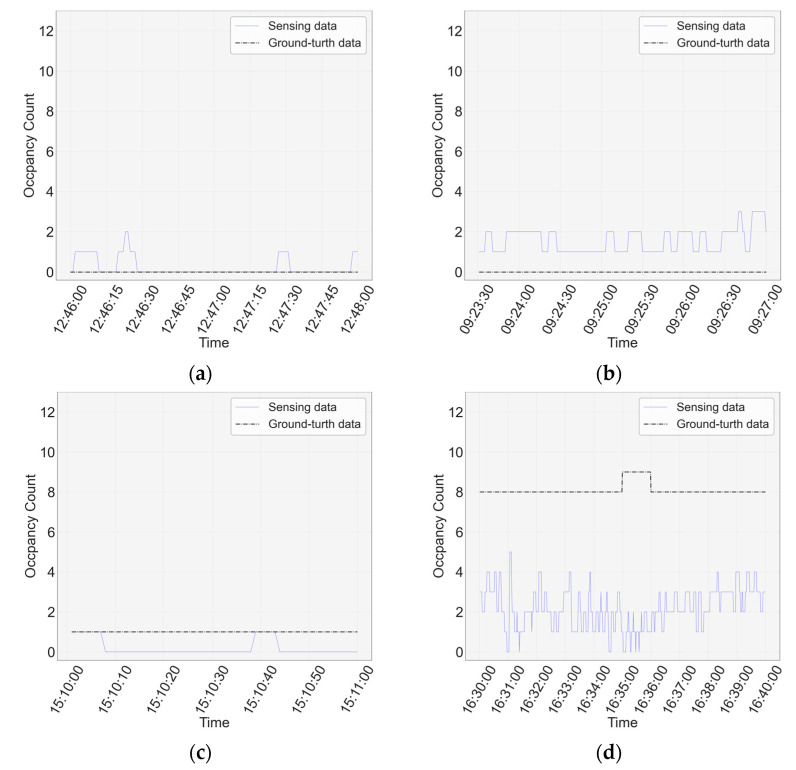
Classification of the occurrence of measurement errors: (**a**) case where light reflection occurs; (**b**) case where an object is recognized as a person; (**c**) case where a person is obscured by an object; (**d**) case where a person is obscured by a person.

Because the type-1 camera is affected by the surrounding environment, measurement errors occur, as shown in Figure 6, Figure 7 and Figure 8. The blue solid line represents the sensing data and the black dotted line represents the ground-truth data. Figure 9a shows the case where light reflection occurs. This measurement error occurs in light reflection owing to the characteristics of the camera; therefore, it can be sensed even when no person is present. Figure 9b shows the case of recognizing an object as a person, which is mainly caused by clothes hanging from the backrest of a chair. Figure 9c shows a case in which a person is covered by an object, and it occurs when a small person is covered by a chair or sleeps face down. In this case, a person may not be sensed even if present. Figure 9d shows a case in which a person obscured another person. It can be observed that the occupancy measurement error is slightly underestimated.

**Figure 10:**
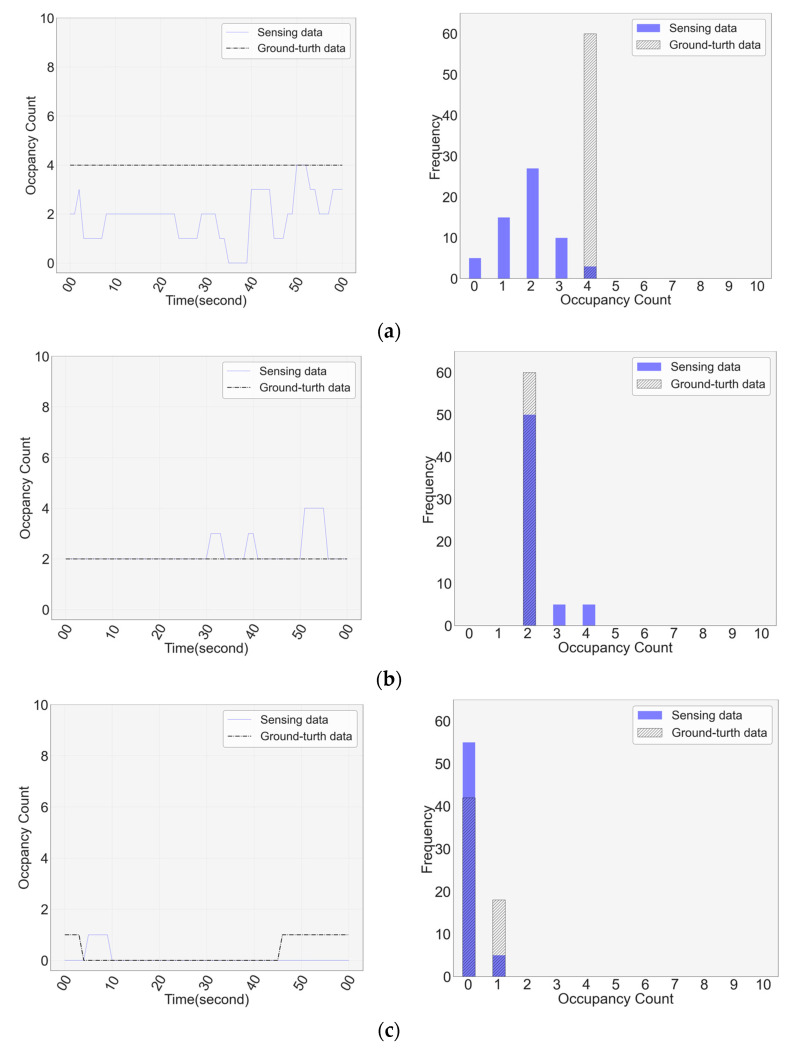
Pattern and histogram of sensing and ground-truth data: (**a**) case where the sensing data fluctuate significantly; (**b**) case where sensing data are partially incorrectly sensed; (**c**) case where ground-truth data have fluctuations.

Figure 10 shows the sensing situation and distribution characteristics during the sensing. In the figure, blue indicates the sensing data, and black indicates the ground-truth data. The left graph is a time-series graph of the situation in which the occupancy count was measured. The *X*-axis represents time, and the *Y*-axis represents the occupancy count. The graph on the right is a distribution graph for the sensing situation; *X*-axis shows the occupancy count, and *Y*-axis shows the frequency in one minute. Figure 10a shows the case in which the sensing data fluctuate significantly, and only some sensing data values match the ground-truth data. In this case, there are several distributions because there are many occupants and there is a lot of movement, and the highest sensing data value coincides with the ground-truth data. Figure 10b shows the case where the sensing data agree with the ground-truth data in most cases; however, some erroneous sensing data exist because of the error occurrence situation, as shown in Figure 9. In this case, the most frequently sensed data value matched the ground-truth data. Figure 10c shows the case in which there is a movement of the population for 1 min, and there is a change in the sensing and ground-truth data. In this case, the sensing data have an error; however, the occupancy count with the highest frequency coincides with the sensing and ground-truth data. Thus, sensing data show various measurement characteristics, and it is difficult to estimate occupancy at a time interval of a second. Therefore, a deep learning-based occupancy estimation was performed using distribution characteristics at a time interval of a minute.

#### 3.2.2. Estimation of Occupancy Using Deep Learning

**Figure 11:**
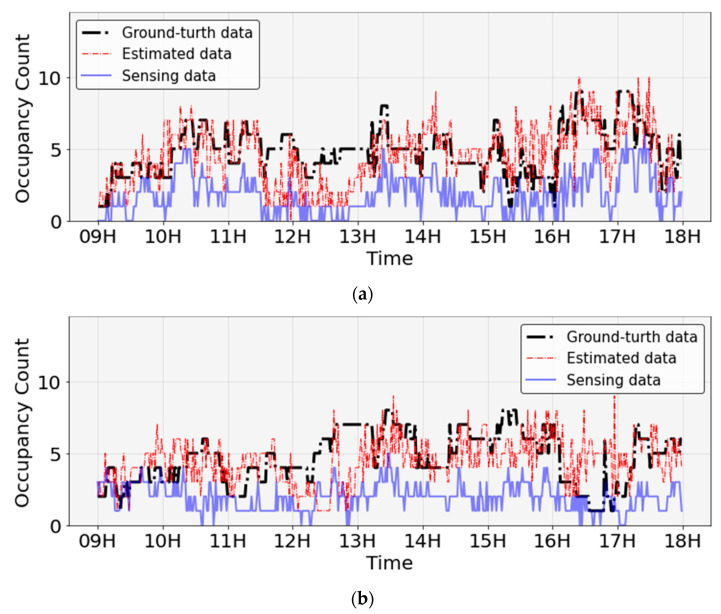
Estimation of occupancy using deep learning: (**a**) room 1; (**b**) room 2.

In this section, the occupancy at a time interval of a minute is estimated using the distribution characteristics of the analyzed sensing data. The occupancy was estimated using the deep learning model and hyperparameter settings described in the previous section. In Figure 11, the black dotted line represents the ground-truth data, the red dotted line represents the estimated data, and the solid blue line represents the sensing data. The sensing data patterns based on the time of rooms 1 and 2 had a strong tendency to be underestimated compared to the ground-truth data. The estimated data were closer to the ground-truth data than to the sensing data for both rooms 1 and 2. In some cases, the estimated data were expected to be higher than the ground-truth data; however, the peak value was expected to be better than the sensing data. In addition, in the estimated data, similar to the trend observed in the ground-truth data, occupancy increased around 9:00, decreased between 12:00 and 13:00, around lunchtime, and decreased after 17:00.

Table 2 shows the evaluation of the estimated occupancy using deep learning. In the case of room 1, the RMSEs of the sensing and estimated data were 3.5 and 1.9, respectively. The estimated data were improved by 84.2% using deep learning. In the case of room 2, the RMSE of the sensing data was 3.0, and the RMSE of the estimated data was 1.9, which was an improvement of 57.9%. The error of the estimated result was reduced compared to that of the sensing data. Therefore, it is desirable to estimate and utilize occupancy in a room using deep learning rather than directly using occupancy from sensing data.

**Figure 12:**
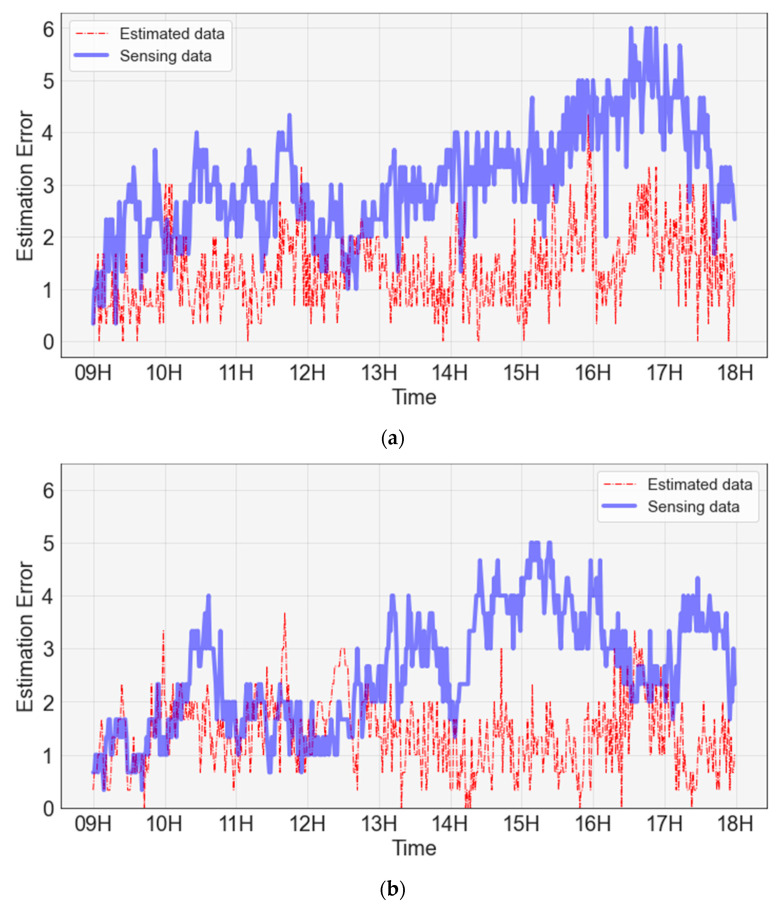
Analysis of occupancy error by time zone between sensing and estimated data: (**a**) average measurement error in room 1; (**b**) average measurement error in room 2.

Figure 12 shows the time-zone error of the sensing and estimated data from the ground-truth data for rooms 1 and 2. In both rooms 1 and 2, the error of the estimated data decreased overall compared with the sensing data. In particular, in the case of room 1, the error between 9:00 and 10:00 was greatly reduced from 2–3 people during sensing to 1–2 people through estimation. In the case of room 2, the error between 14:00 and 18:00 significantly decreased from 3–6 people during sensing to 1–3 people through estimation.

**Figure 13:**
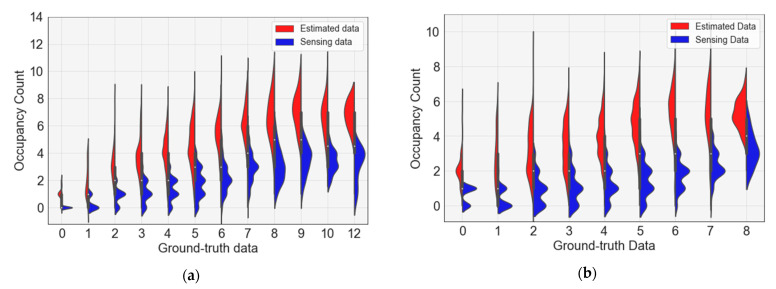
Analysis of occupancy count error distribution between sensing and estimated data: (**a**) occupancy distribution in room 1; (**b**) occupancy distribution in room 2.

Figure 13 shows the error distribution of the occupancy count between the sensing and estimated data for rooms 1 and 2. In the case of sensing data for both rooms 1 and 2, occupancy was often underestimated; however, it was improved through estimation using deep learning. However, when the occupancy in room 1 was more than eight people, both the sensing and estimated data showed a somewhat low accuracy.

### 3.3. Prediction of Energy Consumption

#### 3.3.1. Analysis of Energy Consumption

**Figure 14:**
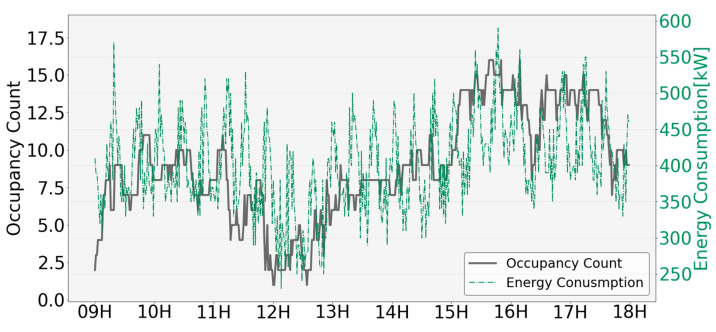
Analysis of occupancy and energy consumption pattern.

In this section, the occupancy (ground-truth, sensing, and estimated data) and energy consumption presented in Phase 3 are analyzed. The occupancy count was calculated by integrating rooms 1 and 2, considering that the energy consumption levels were connected to each other. Figure 14 shows the occupancy of the ground-truth data and energy consumption collected at a time interval of a minute. The green solid line represents the energy consumption data and the solid black line represents the occupancy count. Energy consumption increased for a certain period (approximately 30 min) and then decreased, showing a pattern of consumption that repeated periodically throughout the day. This energy consumption pattern appeared to be a periodic operation characteristic based on the set temperature of the air conditioner because the plug loads in rooms 1 and 2 and the air conditioner in the server room were connected. The energy consumption pattern was similar to that of occupancy, which increased after 9:00, decreased from 12:00 to 13:00, increased again, and decreased after 17:00.

#### 3.3.2. Analysis of the Correlation between Occupancy and Energy Consumption

**Figure 15:**
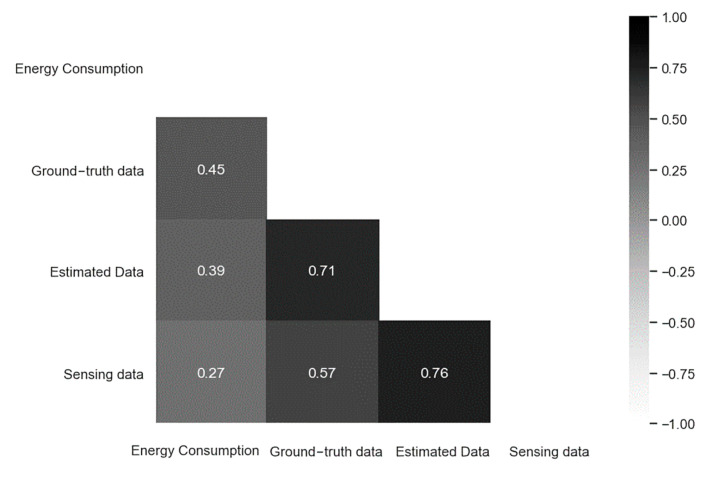
Correlation analysis of occupancy and energy consumption.

The energy consumption for the effect of occupancy was analyzed using PCC. The correlation between occupancy and energy consumption is shown in Figure 15. The correlation between occupancy and energy consumption was highest for ground-truth data at 0.45, followed by estimated data at 0.39, and sensing data at 0.27, which was the lowest. As such, energy consumption has a meaningful correlation with occupancy; therefore, energy consumption will be predicted using occupancy.

#### 3.3.3. Prediction of Energy Consumption Using Deep Learning

**Figure 16:**
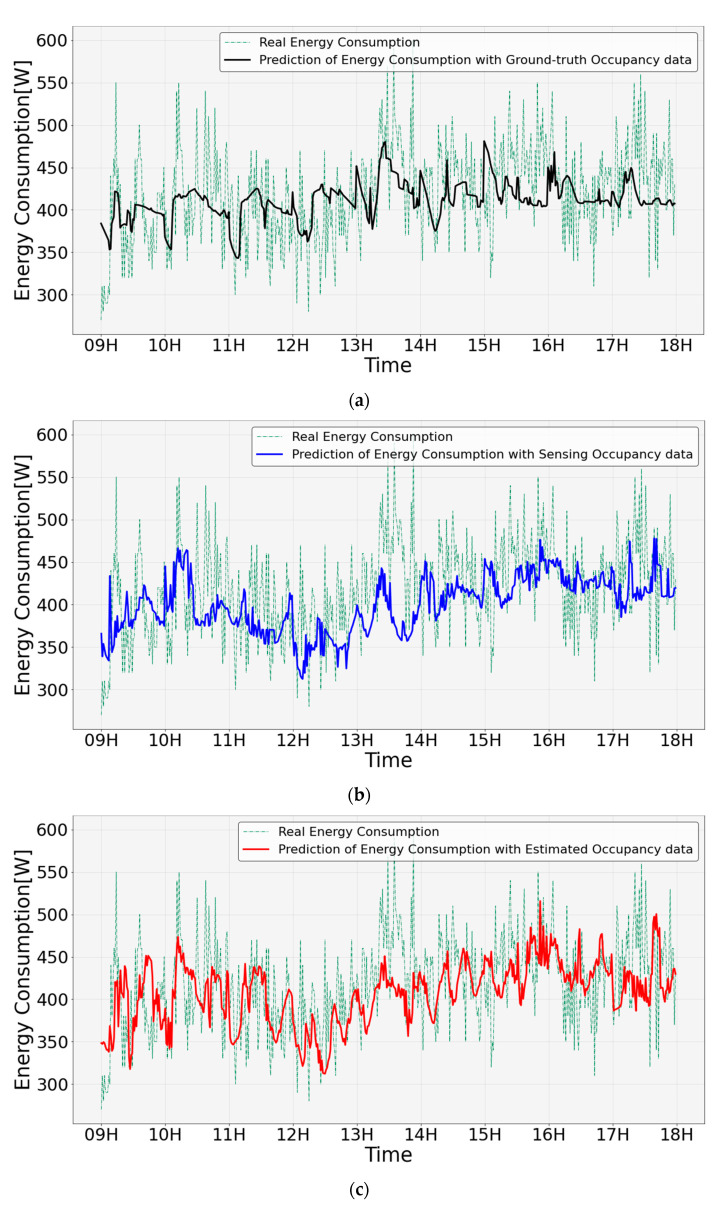
Prediction result of energy consumption based on the occupancy using deep learning: (**a**) prediction of energy consumption with ground-truth data; (**b**) prediction of energy consumption with sensing data; (**c**) prediction of energy consumption with estimated data.

Based on the data analysis mentioned in the previous section, a deep learning-based energy consumption prediction was performed using occupancy. This was performed using the deep learning model and hyperparameter settings described above, and the predicted results are shown in Figure 16. Figure 16a shows the energy consumption data predicted using the actual energy consumption and ground-truth data, Figure 16b shows the energy consumption data predicted using the sensing data, and Figure 16c shows the energy consumption data predicted using the estimated data. The energy consumption prediction of the ground-truth data follows the trend well for all periods compared to the energy consumption predicted using sensing and estimation. The prediction of energy consumption using sensing data tends to be underestimated. The energy consumption prediction by the estimated data also tends to be underestimated; however, it is improved compared to the sensing data and relatively follows the energy consumption trend.

**Figure 17:**
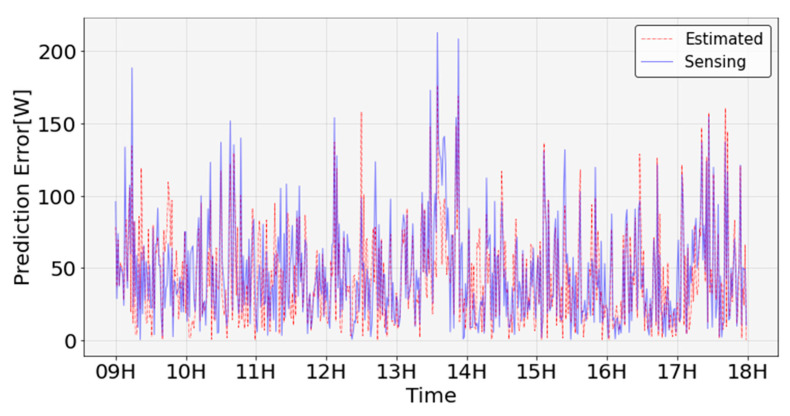
Energy consumption prediction error based on sensing and prediction data.

A time-zone error analysis of the energy consumption predicted using the sensing data and energy consumption predicted using the estimated data was performed, as shown in Figure 17. For both the energy consumption predicted using the sensing data and that predicted using the estimation data, a large prediction error occurred between 13:00 and 14:00. This error was estimated because the peak of the actual energy consumption between 13:00 and 14:00 was not predicted. The numerical analysis showed that the actual energy consumption was 600 W at 13:53. The energy consumption predicted using the estimated data was approximately 430 W, resulting in an error of 170 W. In addition, the energy consumption predicted using the sensing data was approximately 370 W, resulting in an error of approximately 230 W.

Table 3 shows the evaluation of the energy consumption prediction. The RMSE of energy consumption based on the ground-truth data was 5.4, the RMSE of energy consumption based on the estimated data was 5.6, and the RMSE of energy consumption based on the sensing data was 5.9. The estimated data predicted the energy consumption 5.2% more accurately based on RMSE than the sensing data, and it can be observed that the accuracy of the estimated data improved the energy consumption prediction error.

The total energy predicted for a day by occupancy from ground-truth data was 22.2 kWh, which was 0.4 kWh different from the actual energy used. The total energy predicted for a day by occupancy based on the estimated data was 21.2 kWh, which was 1.4 kWh different from the actual energy used. In addition, the total energy predicted for a day by occupancy based on the sensing data was 21.1 kWh, which was 1.5 kWh different from the actual energy. Energy consumption prediction based on the estimation of occupancy can reduce the difference in energy consumption by 5.8% compared with predictions based on sensing occupancy.

## 4. Conclusions

In this study, a deep learning model was developed using ground-truth data to increase the occupancy estimation accuracy. The enhanced occupancy accuracy improved the energy consumption prediction. Errors in the collected data were analyzed, occupancy was estimated, and energy consumption was predicted based on the estimated occupancy using the proposed method. Results showed that the accuracy of the conventional camera-based sensing occupancy was 3.3, on average, based on RMSE, and it was improved by 71.1% to 1.9 using the proposed model. The accuracy of energy prediction using the sensing occupancy was 58.9 based on RMSE. However, the accuracy was improved by using the estimated occupancy; the prediction performance improved by 5.2% compared to the previous instance, which was 56.0.

Although the accuracy of sensors is improving with the development of technology, a sensor inevitably causes measurement errors based on factors such as the communication environment and the installation location. To address this problem, the accuracy of occupancy estimation using sensing data was improved using deep learning in this study. The estimated occupancy had lower accuracy than the ground-truth data but higher accuracy than the sensing data. In addition, the energy consumption prediction accuracies were in the following order: ground-truth, estimated, and sensing. Thus, it can be observed that the accuracy of the energy consumption prediction was improved using the result of the estimated occupancy based on deep learning.

In the camera-based occupancy measurement used in this study, there were errors in the sensing values due to external environmental factors, such as the camera installation location. Therefore, a more detailed consideration of the installation area is required for camera-based occupancy measurements. In this study, occupancy in rooms 1 and 2 was integrated to predict energy consumption. However, better performance was expected if the prediction was performed by configuring the rooms independently. In addition, although the DNN model was used to estimate occupancy and predict energy consumption, there is a plan to expand this model to a deep learning approach that can utilize time-series characteristics. The proposed approach can be used to observe long-term effects and seasonal impacts by performing extended observations. In addition, research needs to be extended using various deep learning models such as DNN, CNN, and LSTM to find appropriate models and improve predictive performance in the future. An ensemble approach that combines multiple models can also be used to enhance performance. Identifying individuals can also improve occupancy accuracy by eliminating double-counting and reducing errors.

## Figures and Tables

**Table 1:** Comparison of previous studies for energy consumption prediction using occupancy.

Reference	Case	Load	Occupancy Sensor	Method	Electricity Consumption	Performance
Ding et al. [19]	Campus Building	HVAC, Plug, Light	PIR	Mathematical	680 W (average)	Within 5%
Wei et al. [20]	Commercial Building	HVAC	CO2	Ensemble	~940 W (average)	RMSE 30
Markovic et al. [21]	Campus Buildings	Plug	PIR	LSTM	365 W (peak)	RMSE 19
					900 W (peak)	RMSE 54
					367 W (peak)	RMSE 82
Anand et al. [22]	Institutional Building	Plug, Light	WiFi	ANN-DN	~500 kW (peak)	RMSE 32.5
Wang et al. [23]	Office Building	Plug	Camera	LSTM	~15 kW (peak)	RMSE 0.42

**Table 2:** Evaluation of estimated occupancy.

	MAE		MSE		RMSE	
	Room 1	Room 2	Room 1	Room 2	Room 1	Room 2
Estimation (Proposed)	1.4	1.5	3.5	3.6	1.9	1.9
Sensing (Conventional)	3.1	2.6	12.3	9.1	3.5	3.0

**Table 3:** Evaluation of energy consumption prediction.

	MAE	MSE	RMSE	CV(RMSE)	MAPE
Ground-truth	4.4	29.6	5.4	13.0%	0.107
Estimation (Proposed)	4.5	31.3	5.6	13.4%	0.107
Sensing (Conventional)	4.7	34.7	5.9	14.1%	0.111

## Data Availability

Not applicable.
